# Sexually dimorphic effects of early life stress in rat pups on urinary bladder detrusor muscle contractility in adulthood

**DOI:** 10.1186/s13293-016-0062-1

**Published:** 2016-01-27

**Authors:** Ehsan Mohammadi, Dawn K. Prusator, Eleanor Healing, Robert Hurst, Rheal A. Towner, Amy B. Wisniewski, Beverley Greenwood-Van Meerveld

**Affiliations:** VA Medical Center, Oklahoma City, OK USA; Department of Physiology, University of Oklahoma Health Science Center, Oklahoma City, OK USA; Oklahoma Center for Neuroscience, University of Oklahoma Health Science Center, Oklahoma City, OK USA; Department of Urology, University of Oklahoma Health Science Center, Oklahoma City, OK USA; Advanced Magnetic Resonance Center, Oklahoma Medical Research Foundation, Oklahoma City, OK USA

**Keywords:** Painful bladder syndrome/interstitial cystitis, Sex differences, Early life stress, Rats, Urinary bladder, Males, Females

## Abstract

**Background:**

Painful bladder syndrome/interstitial cystitis (PBS/IC) is a chronic disorder that is commonly seen in women who report a history of adversity in early life. Here, we test the hypothesis that early life stress (ELS) induces sexually dimorphic abnormalities in urinary bladder smooth muscle function in adulthood.

**Methods:**

Male and female rat pups were conditioned on postnatal (PN) days 8–12 with either a “predictable or “unpredictable” odor-shock, or odor only control treatment. In adulthood, urinary bladder function was assessed in vivo via urine spot analysis and in vitro via contractile responses to electrical field stimulation (EFS) and membrane depolarization with potassium chloride (KCl).

**Results:**

In adulthood, we found that female rats exposed to unpredictable ELS showed a significant (*p* < 0.05) increase in urine voiding volume compared to predictable ELS or controls. We also found that detrusor muscle contractile responses to EFS were significantly (*p* < 0.001) decreased following unpredictable ELS in adult female rats compared to the predictable ELS or controls. In male rats exposed to ELS, there was no difference in voiding volume or EFS-induced contractility between groups. In adulthood, the myogenic smooth muscle response to KCl was not significantly different between groups. Histological analysis from adult female and male rats revealed no differences in the appearance of the urinary bladder in rats exposed to ELS.

**Conclusions:**

In summary, our findings provide evidence to support abnormalities in the nerve-mediated contractile responses of the detrusor smooth muscle in adult female rats following ELS. We speculate that these sexually dimorphic alterations in urinary bladder function may account, at least in part, for the female predominance of PBS/IC.

## Background

Painful bladder syndrome/interstitial cystitis (PBS/IC) affects 100–300 per 100,000 women and has detrimental effects on health-related quality of life [1]. The symptoms of PBS/IC include chronic pelvic pain, pressure and discomfort associated with the bladder and other urinary symptoms such as urgency and increased voiding frequency [2]. Currently, few treatment options are available to treat patients with this debilitating condition, and further studies into the mechanisms of PBS/IC are required to aid in the development of novel drug targets to alleviate the symptoms. There are several factors that may predispose an individual to develop PBS/IC, or may exacerbate existing symptoms in patients with a clinical diagnosis of PBS/IC, these include but are not limited to gender, other non-bladder-related pain syndromes, history of infection, and instances of stress [3, 4]. Gender bias in PBS/IC is apparent, with females being five times more likely to develop this condition, suggesting that women are more vulnerable to the development of PBS/IC [5]. Additionally, PBS/IC is often comorbid with other functional pain disorders such as irritable bowel syndrome (IBS), another female-predominant disorder [6]. Although PBS/IC is becoming more widely characterized clinically, the mechanisms underlying the disorder are poorly understood.

Tremendous interest is evolving in understanding the contributions of stress to urinary bladder dysfunction [7, 8]. In vivo studies have highlighted the role of stress in bladder dysfunction [9], while an in vitro study points to decreases in bladder muscle contractility following an acute stressor in adulthood [10]. Additional evidence suggests that PBS/IC is more frequently reported in patients with a history of early life stress (ELS) such as neglect or abuse during childhood [11]; however, despite these recent advances, there are to our knowledge no studies examining the sex-specific effects of neonatal stress on urinary bladder muscle function in adulthood. Therefore the current series of experiments were specially designed to investigate the effect of ELS on urinary bladder function in adulthood, specifically focusing on the detrusor muscle, the primary muscle responsible for the storage and release of urine. The current study tests the hypothesis that ELS induces abnormalities in urinary bladder function and detrusor muscle contractility in adulthood in a sex-dependent manner.

While several rodent models of ELS exist including neonatal maternal separation [12] and limiting bedding [13], we selected an odor-attachment learning (OAL) rodent model, which was developed primarily to mimic attachment to an abusive caregiver [14]. To ensure survival during the early postnatal days, rat pups form an attachment to the dam based on maternal odor and care regardless of whether the interaction is pleasant or noxious [15]. Male and female neonates repeatedly exposed to predictable odor/shock pairings subsequently develop an attachment or preference for the conditioned “maternal” odor. The ability of rat pups to show a preference for the dam in the presence of noxious stimuli is the basis for the OAL model [16]. Successfully conditioned animals move toward the odor they associate with a mild electrical shock when assessed in a Y-maze [14, 15]. Following the OAL exposure, the animals then mature to adulthood allowing us to investigate the potential effect of an adverse early life experience on bladder function in adulthood. We have previously shown that this OAL model induces female-specific colonic hypersensitivity in adult rats and is being used to investigate the relationship between ELS and symptomatology in females with irritable bowel syndrome, a common comorbidity of PBS/IC [17].

Here we report that following neonatal early life conditioning, adult female rats exposed to unpredictable, but not predictable, neonatal ELS exhibited urinary bladder smooth muscle dysfunction. However, in adult male rats exposed to ELS, urinary bladder smooth muscle function resembled their non-stressed counterparts. This study is the first to show sex-related differences in urinary bladder function following unpredictable ELS and provides a platform for investigating the mechanisms by which ELS leads to bladder dysfunction in adult females.

## Methods

### Ethical approval

The “Principles of laboratory animal care” (NIH publication No. 86–23, revised 1985) were followed in accordance with standards established by the *Guide for Care and Use of Laboratory Animals* (1996). All animal experimental protocols were approved by the Veterans Affairs (VA) Institutional Animal Care and Use Committee (IACUC) Protocol # 1191–001.

### Animals

Timed pregnant Long-Evans female rats (*n* = 10) were purchased from Charles Rivers Laboratories (Wilmington, MA). The rats were single-housed under standard rodent conditions with a 12:12 h light-dark cycle. Food and water were made available ad libitum. Female rats arrived approximately on gestational day 9 to allow for acclimation to the animal facility before parturition. The day of birth was assigned as postnatal (PN) day 0, and litters were cross-fostered and culled to no more than 12 pups on PN1. Male and female rat pups from each litter were subjected to the neonatal stress paradigm. All rat pups were then weaned and weighed on PN21 and separated according to sex and treatment into standard rodent cages with two animals per cage. All further experimentation occurred in adulthood ~PN90 using both male and female rats. In all adult rats, the urinary bladder function was assessed in vivo via daily urinary voiding analysis. In separate adult rats, bladder tissue was then isolated and strips of detrusor muscle were mounted in organ baths to assess smooth muscle contractility in response to intramural nerve stimulation (frequency 2–16 Hz, pulse duration 0.5 ms, train duration 5 s, voltage 90 V) and membrane depolarization with high concentrations of KCl (150 mM).

### Neonatal stress paradigm: odor-shock conditioning

The methodology is described in detail in our recent publication [17]. Briefly, neonatal Long-Evans rat pups received a single series of odor-shock conditioning from PN8 to PN12. Each conditioning series consisted of 11 separate, 30-s peppermint odor presentations with a 4-min inter-stimulus interval [14, 17, 18]. The peppermint odor was presented to the animals via a flow dilution olfactometer (Med Associates, Georgia, VT) at a rate of 2 l/min (1:10 concentration of odor). Three groups were conditioned: predictable odor shock, unpredictable odor shock, and odor only. In the predictable odor-shock group, the pups received a 0.5-mA shock (Coulbourn Instruments, Whitehall, PA) at the base of the tail during the final second of the peppermint odor presentation. Rat pups in the unpredictable odor-shock group received the 0.5-mA shock at the base of the tail 2 min after the odor presentation, which did not allow for an association to be made between the odor and the shock. The odor only control group received a 30-s odor presentation in the absence of any shock.

### Neonatal behavioral testing

As described in detail in our recent publication [17], the total behavioral activation was calculated as the average of each trial over all 5 days of conditioning. Odor-shock association learning was assessed by recording the behavioral activation of the pups during each of the 11 conditioning trials from PN8–12. Behavioral activation was measured using a 0 to 5 scale where 0 represents no movement and 5 represents movement of all four limbs and the head [14, 18, 19]. Twenty-four hours following the final conditioning series, odor preference was assessed using a Y-maze test, where pups were given the choice of either peppermint odor or clean bedding. Odor preference was determined by recording the number of choices toward the conditioned peppermint odor out of five trials.

### Urinary voiding analysis in adult rats

In adulthood, urinary bladder function was assessed in freely moving rats from all experimental groups using urine voiding spot analysis following the methodology of Kong and colleagues with minor modifications [20]. Briefly, rats were placed for 1 h into a regular animal facility rodent cage (40 cm × 20 cm × 20 cm) lined with filter paper under normal laboratory lighting. Urine-covered regions were detected with UV light, marked and assessed for any urine spotting patterns. Micturition volume was quantified using ImageJ software to assess surface area covered by urine, and the surface area was then converted to volume. The standard curve for converting pixel area to volume was pixel area (cm^2^) = 88.05(volume [ml]), *r*^2^ = 0.979.

### In vitro measurements of bladder detrusor muscle contractility

Adult rats were anesthetized with isoflurane gas (5 %) and euthanized by decapitation according to approved guidelines. The protocol for the in vitro measurements of bladder detrusor muscle contractility was followed according to the methodology described in our previous study [21]. Briefly, the bladder was removed and placed in ice-cold Krebs solution composed of 120 mM NaCl, 6 mM KCl, 1.2 mM MgCl_2_, 1.2 mM H_2_PO_4_, 2.5 mM CaCl_2_, 14.4 mM NaHCO_3_, and 11.5 mM glucose, aerated with 95 % O_2_–5 % CO_2_. Bladder detrusor muscle strips ~9 mm long and ~2 mm wide with an intact urothelium were prepared and then placed between zigzag electrodes (Radnoti Glass Technology, Monrovia, CA) and mounted in 10-ml organ baths (Radnoti Glass Technology, Monrovia, CA) containing aerated Krebs solution. Detrusor muscle strips were stretched to optimal tension (1 g) and allowed to equilibrate for 30 min. Isometric contractions were recorded in response to graded electrical field stimulation (EFS) from 2, 4, 8, to 16 Hz (pulse duration 0.5 ms, train duration 5 s, and voltage 90 V). Following the EFS protocol, the peak contractions in response to KCl (150 mM) were measured to assess whether ELS had any effect on “myogenic” or receptor-independent detrusor muscle contractility. All muscle activity was recorded on Labchart recording software (AD Instruments, Castle Hill, Australia).

### Bladder histology

In a separate subgroup exposed to ELS, animals were euthanized and the bladder was isolated and placed in 10 % formalin. The fixed tissue was then paraffin-embedded, sectioned at 10 μm, and stained using hematoxylin and eosin (H&E) staining (Precision Histology, Oklahoma City, OK). Stained sections were observed with a Nikon Optophot microscope (Nikon Instruments, Melville, NY) by a board certified animal pathologist blinded to the treatments for signs of damage and inflammatory infiltrate. Images were taken using a Canon EOS Rebel T3i camera (Canon, U.S.A. Inc. Melville, NY).

### Data analysis

Data is shown as mean ± SEM. Learning acquisition data was examined for statistical significance using a two-way repeated measures analysis of variance (ANOVA) for behavioral activation and a one-way ANOVA for odor preference in the Y-maze followed by a Bonferroni post hoc test. Body weight data was analyzed separately for neonatal or adult rats via two-way ANOVA followed by a Bonferroni post hoc test to identify specific differences between sex and treatments. Urine voiding volume data was analyzed via two-way ANOVA followed by a Bonferroni post hoc test to identify specific differences between sex and treatments. Data collected from organ bath experiments was calculated as change in force from baseline tension at 1.0 g and expressed as force per square centimeter of tissue length. To determine statistical significance between multiple control and treatment groups, EFS data was compared using separate two-way repeated measures ANOVA followed by a Bonferroni post hoc test (for each ELS treatment, sex × frequency; for each sex, ELS treatment × frequency). For KCl response graphs, two-way ANOVA followed by a Bonferroni post hoc test (sex and treatments) was applied. Results were deemed significant when *p* values were less than 0.05 (Graphpad Prism 6.0c; La Jolla, CA). Data from two animals were lost during urine voiding assay due to extensive chewing of the filter paper. Lack of response of the detrusor muscle strip to EFS and KCl in three animals resulted in eliminating them from data analysis.

## Results

### Rodent model of adverse early life experience

**Fig. 1:**
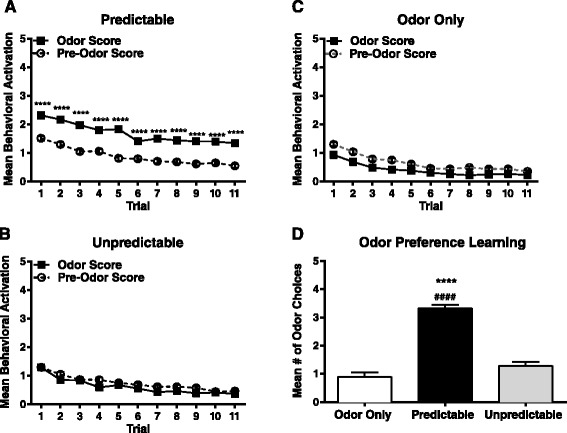
Neonatal learning acquisition in response to classical conditioning. Neonatal behavioral activation was recorded during each trial from PN8–12 during odor presentation for pups experiencing **a** predictable odor-shock (*n* = 24), **b** unpredictable odor-shock (*n* = 28), or **c** odor only control treatment (*n* = 27), and compared to their pre-stimulus score on that same day. Animals experiencing predictable odor-shock conditioning exhibit increased behavioral activation during odor presentation whereas the unpredictable or odor only control animals exhibit no increases in behavioral activation during odor presentation. **d** Odor preference analysis using the Y-maze shows that pairing of the odor and shock stimuli created an odor preference in the predictable group for the conditioned odor compared to either the unpredictable (####*p* < 0.0001) or odor only controls (****p < 0.0001) who showed an absence of odor preference

Following predictable odor-shock conditioning, analysis of behavioral activation revealed a significant main effect of trial (*F*(10,230) = 30.14, *p* < 0.0001) and odor presentation (*F*(1,23) = 108.9, *p* < 0.0001) during neonatal conditioning. Animals that underwent predictable odor-shock conditioning exhibited increased behavioral activation during the 30-s peppermint odor presentation (*p* < 0.0001) indicating a learned association between the odor and the aversive stimuli (Fig. 1a). The pups in the odor only control and unpredictable odor-shock conditioning groups showed no differences in behavioral activation upon exposure to the odor, which indicates that no association was made between the odor and the aversive stimuli (Fig. 1b, c). Y-maze testing revealed a significant effect of treatment on odor choice (*F*(2,76) = 78.51, *p* < 0.0001) wherein animals in the predictable group showed a significant preference for the peppermint odor on the Y-maze test compared to animals who experienced unpredictable ELS (*p* < 0.0001) or odor only controls (*p* < 0.0001). The unpredictable and odor only pups did not display a preference for either odor (Fig. 1d). The odor preference data confirms that animals in the predictable odor-shock group learned an attachment to the conditioned peppermint odor in conjunction with the associated aversive stimulus.

### Body weight

Following ELS exposure, all animals were weaned and weighed; while there was a significant main effect of sex (*F*(1,73) = 4.03, *p* = 0.048), no difference in weaning weight was observed between male or female treatment groups compared to their respective odor only controls (Table 1). In adulthood, animals were weighed at PN90, and while the main effect of sex was highly significant (*F*(1,73) = 260.5, *p* < 0.0001), the ELS treatment groups exhibited no differences in body weight (Table 1). These data indicate that ELS exposure did not alter growth during early or adult life.

**Table 1:** There were no differences in body weight between male and female treatment groups compared to their respective odor only controls in neonates and adults

Treatment groups	Neonates		Adults	
	Body weight (g)		Body weight (g)	
	Female	Male	Female	Male
Odor only (*n* = 27)	60 ± 1	62 ± 1	355 ± 11	570 ± 21
Predictable ELS (*n* = 24)	59 ± 1	60 ± 2	314 ± 9	563 ± 18
Unpredictable ELS (*n* = 28)	61 ± 2	64 ± 1	355 ± 16	574 ± 22

Value shown as mean ± SEM

### The effect of ELS on urine voiding in adult rats

**Fig. 2:**
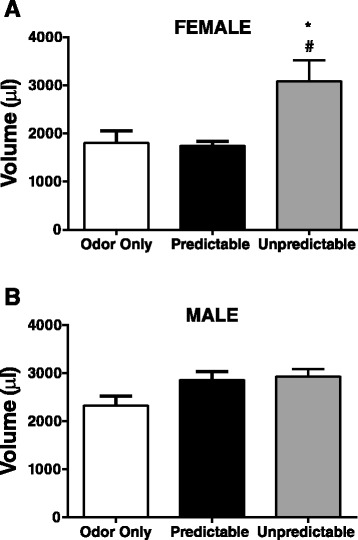
Urinary voiding in adult female and male rats following ELS exposure. Urinary voiding volume was assessed using urine spot analysis in 20 adult female (**a**) and 18 male (**b**) rats. Following unpredictable ELS, 7 females exhibit increased voiding volumes indicated by an increased area of urine saturation compared with 5 predictable odor-shock (#*p* < 0.05) and 8 odor only control females (*p < 0.05). **b** In male rats, a history of unpredictable early life adverse experience (6 rats) did not alter voiding volumes compared to males exposed to predictable ELS (5 rats) or odor only controls (7 rats)

In adulthood, urine spot analysis was used to assess urinary bladder function in freely moving adult rats previously exposed to early life conditioning. Two-way ANOVA demonstrated a significant main effect for sex (*F*(1,32) = 4.96, *p* = 0.033) and ELS treatment (*F*(2,32) = 7.03, *p* < 0.01), without a significant interaction (*F*(2,32) = 2.49, *p* = 0.099). Post hoc comparisons demonstrated that in females, unpredictable neonatal experience significantly increased voiding volume in adulthood compared to predictable ELS (*p* < 0.05) and odor only controls (*p* < 0.05) (Fig. 2a). In contrast, there were no post hoc differences in voiding volume between ELS treatments in male rats (Fig. 2b). An assessment of the patterns of urine spotting revealed no consistent patterns of voiding behavior between the experimental groups.

### Sex-related differences on adult bladder detrusor muscle contractility induced by neural stimulation follow ELS

**Fig. 3:**
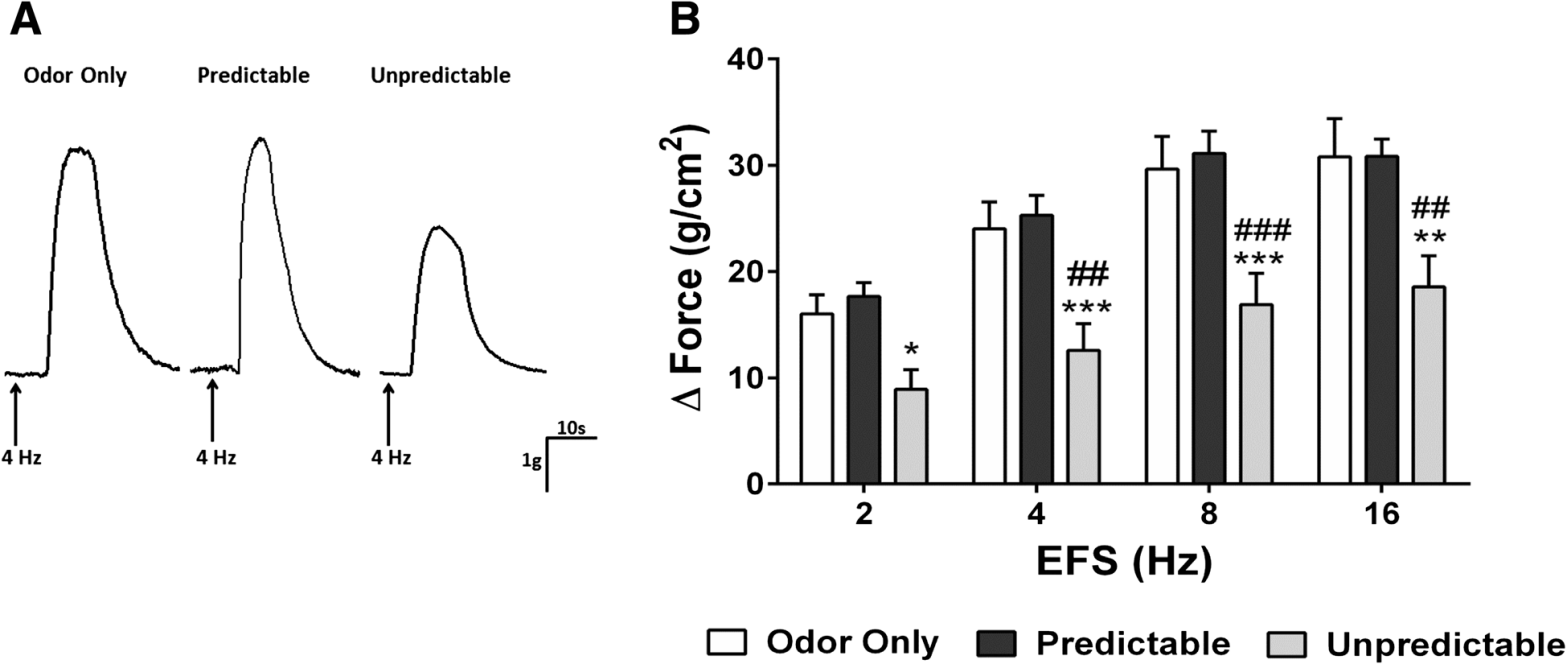
EFS-induced bladder detrusor muscle contractility in adult female rats following ELS. **a** Representative traces of nerve-mediated detrusor urinary bladder contractility in response to EFS (4 Hz) in adult female rats following neonatal conditioning. **b** In the bladder detrusor muscle isolated from adult female rats following neonatal adversity, EFS caused an increase in the amplitude of the bladder detrusor muscle contractile response that was frequency-dependent. In adult female rats that experienced unpredictable odor-shock conditioning (*n* = 6), there was a marked attenuation in the nerve-mediated bladder detrusor muscle contractility compared to odor only control treatment (*n* = 6) or neonatal predictable odor-shock (*n* = 6), (#*p* < 0.05, ##*p* < 0.01, ###*p* < 0.001) and odor only controls (^**^ *p* < 0.01, ^***^ *p* < 0.001). (*n* value is shown as the number of animals)

**Fig. 4:**
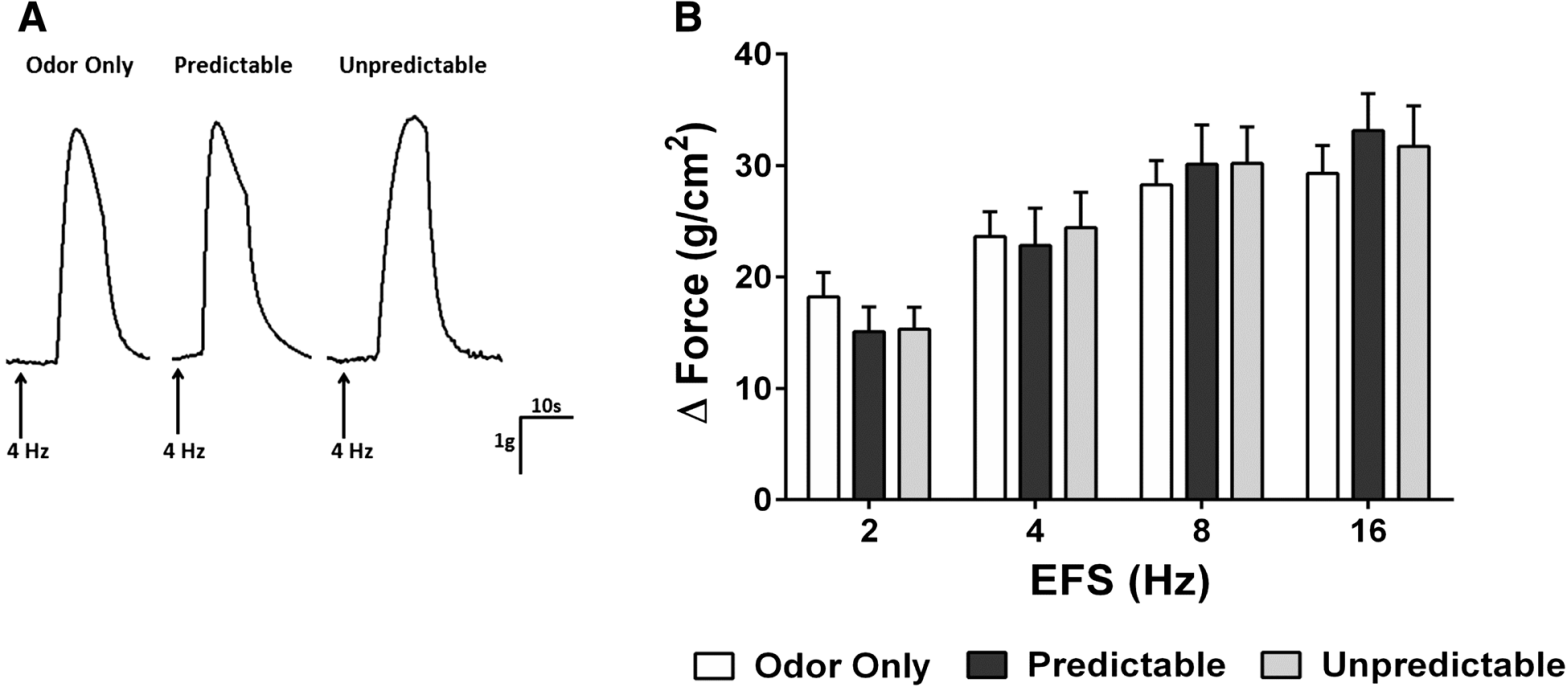
EFS-induced bladder detrusor muscle contractility in adult male rats following ELS. **a** Representative traces of nerve-mediated detrusor urinary bladder contractility in response to EFS (4 Hz) in adult male rats following neonatal conditioning. **b** In the bladder detrusor muscle isolated from adult male rats following neonatal adversity, EFS caused a frequency-dependent increase in the amplitude of the bladder detrusor muscle contractile response. In adult male rats experiencing neonatal predictable odor shock (*n* = 6), or unpredictable odor shock (*n* = 6), there was no differences in the amplitude of the bladder detrusor muscle contractility response compared to odor only controls (*n* = 6). (*n* value is shown as the number of animals)

While there was a significant main effect for EFS frequency, the rats exposed to odor only or predictable ELS treatment did not demonstrate a significant main effect of sex (odor only, *F*(1,18) = 0.006, *p* = 0.93; predictable, *F*(1,18) = 0.08, *p* = 0.78). In contrast, rats exposed to unpredictable ELS demonstrated significant main effects for sex (*F*(1,18) = 8.43, *p* < 0.01), frequency (*F*(3,54) = 75.21, *p* < 0.0001), and interaction (*F*(3,54) = 5.83, *p* < 0.01). In the female rats, representative examples of the EFS contractile responses in all three experimental groups are illustrated in Fig. 3a. In adult females, there was significant interaction between treatment and frequency (*F*(6,81) = 2.93, *p* < 0.05), a main effect of treatment (*F*(2,27) = 8.13, *p* < 0.01) and a main effect of frequency (*F*(3,81) = 139.6, *p* < 0.0001) on detrusor smooth muscle responses to EFS. As shown, EFS responses were significantly decreased following unpredictable ELS compared to the predictable ELS or odor only control groups (Fig. 3a, b). In adult males, analysis revealed a significant effect of frequency (*F*(3,81) = 86.09, *p* < 0.0001) on detrusor muscle contractility; however, exposure to ELS did not induce differences in detrusor smooth muscle contractility in response to EFS (Fig. 4a, b).

### Effect of ELS on detrusor muscle contractility in response to receptor-independent membrane depolarization

**Fig. 5:**
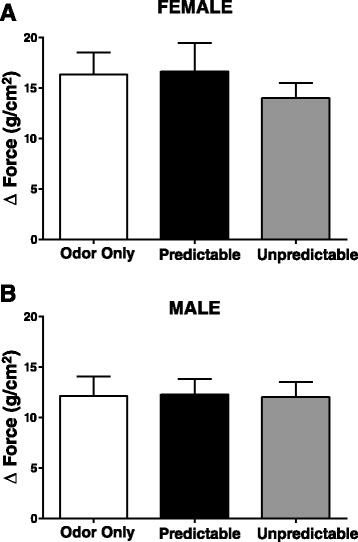
KCl-evoked detrusor muscle contractile response amplitude in female and male rats following neonatal conditioning. Myogenic detrusor muscle contractile responses were elicited using KCl. **a** In adult female or **b** adult male rats following neonatal ELS, the ability of adult bladder detrusor muscle to contract in response to KCl was unaffected by ELS treatment. (*n* value is the same as Fig. 4)

To provoke detrusor muscle contractions via receptor-independent membrane depolarization, we examined the smooth muscle contractile response to KCl. Although there was a significant main effect of sex (*F*(1,54) = 4.76, *p* = 0.034) on KCl-evoked detrusor muscle contractility, there was no significant main effect of ELS (*F*(2,54) = 0.31, *p* = 0.73) on KCl-induced contractility, suggesting that ELS has no effect on the myogenic ability of the bladder detrusor muscle to contract (Fig. 5a, b).

### The effect of ELS on bladder histology

**Fig. 6:**
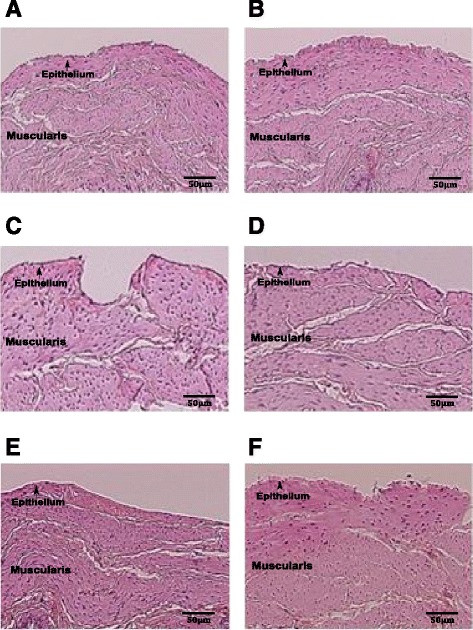
Urinary bladder detrusor muscle histology in adulthood. Histological examination of urinary bladder tissues with H&E staining showed marked similarities in the tissue integrity and gross histological appearance in adult rats. Images show ×5 magnification from representative urinary bladder cross sections from adult rats. **a** Female odor only control; **b** male odor only control; **c** female predictable ELS; **d** male predictable ELS; **e** female unpredictable ELS; **f** male unpredictable ELS

An important question to address is whether neonatal ELS induced changes in the bladder at the time of the contractility studies in the adult animals such as damage or inflammation that could lead to alterations in bladder function. As illustrated in Fig. 6a–f, there was no difference in the gross histological appearance of the tissue in either female or male rats from all three experimental groups. Histology revealed no evidence of inflammation or defects in the urothelium of adult rodents following ELS exposure. Additionally, there was no indication of bladder muscle hypertrophy across groups as defined histologically.

## Discussion

In this study, we provide experimental evidence for sexually dimorphic effects of ELS on urinary bladder function in adult rats. In adult females, we revealed profound changes in urinary bladder voiding following unpredictable ELS compared to those exposed to predictable ELS or odor only controls. In support of a potential mechanism for the abnormal voiding function, we observed a reduction in the amplitude of the contractile responses to EFS in strips of urinary bladder detrusor muscle isolated from adult female rats that had been previously exposed to unpredictable ELS. In contrast to unpredictable ELS, we found that a history of predictable ELS in adult female rats resulted in voiding behavior and nerve-mediated detrusor muscle responses that were similar to those measured in controls not exposed to ELS. In males, exposure to either predictable or unpredictable ELS did not alter adult voiding behavior in vivo or detrusor muscle contractility in vitro. Our findings provide experimental evidence for a sexually dimorphic effect of ELS on urinary bladder function in adulthood that is dependent on the predictability of the early life experience. Taken together, these results are remarkable as they highlight the long-term and complex consequences of ELS on voiding behavior and bladder muscle activity in adulthood that is only evident in female rats following an unpredictable early life adverse experience.

A novel aspect of this study was the use of an experimental model of odor-attachment learning to explore the relationship between the context of childhood adversity and bladder abnormalities in adulthood. Other commonly used rat models of ELS include limiting the bedding material required for the dam to build her nest, which can be equated to a poverty situation [22, 23], or separating the rat pups from the dam in an attempt to model parental neglect [12, 24, 25]. Both experimental paradigms rely on causing stress to the rat dam, which is transferred to the pups via changes in maternal behavior [13]. In contrast, the odor-attachment model utilized in our study has a number of pivotal advantages over previous models of ELS including the fact that the model places direct stress on the neonatal rat pups in an attempt to mimic attachment to an abusive caregiver and the model has the ability to introduce the element of a predictable versus an unpredictable stress in early life, which is an important component of ELS in children [26]. Our previous finding employing the odor-attachment model has shown a female susceptibility to increased visceral pain in adulthood following unpredictable ELS [17], suggesting that the experimental model has both face and construct validity since PBS/IC primarily affects females.

A pivotal strength of this study is the comparison of in vivo voiding behaviors with the in vitro detrusor muscle contractility studies in which we are able to provide a possible explanation for the underlying mechanism for altered detrusor muscle contractility. In vivo, in freely moving rats, analysis of urinary voiding volume revealed that adult males have greater voiding volumes compared to their age-matched female counterparts likely due to their larger adult weight. However, in adult female rats, unpredictable adverse early life experience significantly increased voiding volume suggesting abnormalities in bladder physiology when compared to female rats exposed to predictable ELS or odor only as neonates. Although the stage of the estrus cycle was not monitored in the current study, it is unlikely that the differences in voiding behavior following unpredictable ELS are due to estrus cycle variations since cycling female rats were employed in all of our experimental groups, and variability of female data was not greater than that within male data sets. Furthermore, previous studies have shown that estrus cycling does not influence micturition [27]. Another potential concern in our data interpretation is that the neonatal ELS conditioning may have affected the micturition reflex since the ELS conditioning procedure occurred before complete maturation of the micturition reflex [28]. However, in the current study, we did not observe any differences in the time to void without assistance from the dam between neonatal rats exposed to ELS and controls suggesting no effect of the neonatal conditioning on the micturition reflex.

Building upon our observations that ELS affects voiding behavior in adult female rats, we sought to investigate further these sex-specific alterations in urinary bladder function. In isolated urinary bladder detrusor muscle from adult female rats exposed to unpredictable neonatal conditioning, we found a significant decrease in nerve-mediated detrusor muscle contractility. These findings suggest that changes in bladder detrusor muscle contractility initiated by an adverse early life experience involve changes in the innervation of the urinary bladder smooth muscle. Of importance to this interpretation, the abnormalities in urinary bladder detrusor muscle contractility observed in female rats following unpredictable ELS were evident in the absence of any alterations in bladder smooth muscle thickness, as shown histologically, or the myogenic smooth muscle response induced by receptor-independent membrane depolarization of the detrusor muscle with KCl. Although there were sex-dependent increases in the amplitude of bladder detrusor muscle following KCl administration, the contractility responses were not significantly different between control and treatment groups in our ELS paradigm, indicating that ELS has no effect on the ability of the bladder detrusor muscle to contract via receptor-independent membrane depolarization. Therefore, we are confident that changes in bladder detrusor muscle contractility are due to changes within the neuronal circuitry innervating the bladder musculature. However, since EFS activates both sensory and motor components of the muscle innervation, using the current experimental paradigm, we are unable to distinguish differences in the sensory versus the motor innervation of the bladder smooth muscle following unpredictable ELS. The abnormal smooth muscle contractility observed in this study complements our previous work showing a reduction in detrusor muscle contractility following an acute stressor in adulthood [21], thus further indicating a role for stress in altered detrusor muscle contractility [10]. Taken together, these findings suggest that whether the stressful event occurs in early life or acutely in adulthood, a common mechanism involving innervation of the bladder smooth muscle may lead to abnormalities in urinary bladder muscle function. Knowledge of neuronal plasticity that occurs following ELS remains a fundamental question and is beyond the scope of the current work. However, our future studies will attempt to elucidate the changes in neuronal phenotype that accompany urinary bladder dysfunction following ELS by investigating specific deficits in post-synaptic receptor expression (e.g., muscarinic or purinergic receptors in the muscle).

Another important observation in our data interpretation was the absence of differences in the histological appearance of the detrusor muscle or evidence of inflammatory infiltrate between rats exposed to neonatal ELS and controls. These results imply that bladder dysfunction following ELS does not result from changes in muscle thickness or a chronic inflammatory insult. However, it is possible that mast cell degranulation may have contributed to abnormal bladder detrusor muscle contractility [29]. Exploring the importance of mast cells in urinary bladder detrusor muscle dysfunction following unpredictable ELS represents an avenue for future research endeavors.

## Conclusions

In summary, using a novel experimental model in which neonatal rats were exposed to different pairings of an odor and shock to control for trauma predictability, this study explored the hypothesis that stress in early life produces sex-related abnormalities in nerve-mediated urinary bladder contractility in adulthood. We found that adult female rats exposed to unpredictable neonatal ELS exhibited urinary bladder smooth muscle dysfunction in vivo and in vitro compared to their male counterparts, and to the best of our knowledge, this is the first report of sex-related differences in urinary bladder function following neonatal adversity. Our findings have shown that the nature of the early life experience serves as a strong predictor of bladder dysfunction later in life and advance our understanding of patients with a history of ELS that complain of bladder dysfunction.

## Abbreviations

EFS: electrical field stimulation
ELS: early life stress
OAL: odor-attachment learning
PBS/IC: painful bladder syndrome/interstitial cystitis
PN: postnatal
